# Profiles of Organic Food Consumers in a Large Sample of French Adults: Results from the Nutrinet-Santé Cohort Study

**DOI:** 10.1371/journal.pone.0076998

**Published:** 2013-10-18

**Authors:** Emmanuelle Kesse-Guyot, Sandrine Péneau, Caroline Méjean, Fabien Szabo de Edelenyi, Pilar Galan, Serge Hercberg, Denis Lairon

**Affiliations:** 1 Sorbonne Paris Cité Research Center, Unité de Recherche en Epidémiologie Nutritionnelle, Inserm (U557); Inra (U1125), Cnam, Université Paris 13, Bobigny, France; 2 Département de Santé Publique, Hôpital Avicenne, Bobigny, France; 3 Aix Marseille Université, NORT; Inserm, UMR S 1062; Inra, UMR INRA 1260, Marseille, France; CRCHUM-Montreal Diabetes Research Center, Canada

## Abstract

**Background:**

Lifestyle, dietary patterns and nutritional status of organic food consumers have rarely been described, while interest for a sustainable diet is markedly increasing.

**Methods:**

Consumer attitude and frequency of use of 18 organic products were assessed in 54,311 adult participants in the Nutrinet-Santé cohort. Cluster analysis was performed to identify behaviors associated with organic product consumption. Socio-demographic characteristics, food consumption and nutrient intake across clusters are provided. Cross-sectional association with overweight/obesity was estimated using polytomous logistic regression.

**Results:**

Five clusters were identified: 3 clusters of non-consumers whose reasons differed, occasional (OCOP, 51%) and regular (RCOP, 14%) organic product consumers. RCOP were more highly educated and physically active than other clusters. They also exhibited dietary patterns that included more plant foods and less sweet and alcoholic beverages, processed meat or milk. Their nutrient intake profiles (fatty acids, most minerals and vitamins, fibers) were healthier and they more closely adhered to dietary guidelines. In multivariate models (after accounting for confounders, including level of adherence to nutritional guidelines), compared to those not interested in organic products, RCOP participants showed a markedly lower probability of overweight (excluding obesity) (25≤body mass index<30) and obesity (body mass index ≥30): −36% and −62% in men and −42% and −48% in women, respectively (P<0.0001). OCOP participants (%) generally showed intermediate figures.

**Conclusions:**

Regular consumers of organic products, a sizeable group in our sample, exhibit specific socio-demographic characteristics, and an overall healthy profile which should be accounted for in further studies analyzing organic food intake and health markers.

## Introduction

During FAO international conference held in 2010 [1], a global definition of sustainable diets was proposed: “Sustainable diets are those diets with low environmental impact which contribute to food and nutrition security and to a healthy life for present and future generations. Sustainable diets are protective and respectful of biodiversity and ecosystems, culturally acceptable, accessible, economically fair and affordable; nutritionally adequate, safe and healthy, while optimizing natural and human resources”. In the light of this definition, it is clear that a major challenge exists for nutrition specialists and health care workers [2]. In most industrialized countries, it is widely recognized that current lifestyle and dietary patterns, particularly energy-dense diets rich in saturated fats and added sugars, are not optimal for sustaining health [3], [4]. Indeed, these lifestyles are at least partly responsible for the growing rates of overweight and obesity, which are in turn associated with the increasing prevalence of chronic diseases such as metabolic syndrome, type 2 diabetes, cardiovascular diseases and some cancers [3], [4].

In most countries, a small fraction of farmers and the general population have long shown great concern about this question. Indeed, facing the changes that have taken place in the food production system, refusal of chemical fertilizers, pesticides and intensive animal husbandry since the 1970’s, gave rise to so-called “organic”, “biological”, “biodynamic” and “agro-ecological” productions, depending on the options and/or the country. These alternative production systems are now being recognized because of their low environmental impact [5] and are being certified according to specific regulations and labels in most countries and continents. Such organic production has markedly increased during the last decade, representing up to 3–20% (mean 5.1%) of agricultural acreage in European Union countries, but only 0.6% in the USA [6]. This has been largely driven by consumer attitudes and the growing demand for specific foodstuffs, with a yearly increase of over 10%, reaching, in 2010, a worldwide production of 700 million tons of food per year and a market share of about 60 billion US $/year [7]. In 2010, the countries with the largest markets were the United States, Germany and France [6].

In this context, a diet based on organic products may better meet the definition of sustainability. From a public health point of view, it is thus crucial to understand and analyze organic-product-related consumer profiles. Indeed, while the number of consumers of organic food is markedly rising, limited knowledge is available regarding the nutritional interest and safety of organic food [8]–[11]. Moreover, only small-scale studies have described the profiles of organic consumers [12]–[17] and little information is available regarding their actual food and nutrient intakes [18] or diet-related health indicators [19]–[21].

Thus, within the framework of the web-based large ongoing Nutrinet-Santé Cohort Study [22], already including about 104,000 participants by the end of 2011, we sought here to describe the socio-demographic profiles of organic food consumers, along with their food and nutrient intakes and anthropometric characteristics.

## Materials and Methods

### Population

We analyzed data from the Nutrinet-Santé Study, a large web-based prospective observational cohort launched in France in May 2009 with a scheduled follow-up of 10 years (recruitment planned over a 5-year period) that is attempting to investigate the relationship between nutrition and health as well as determinants of dietary behavior and nutritional status. The design, methods and rationale of the Nutrinet-Santé Study have been described in detail elsewhere [22]. Briefly, the study was implemented in a general population and is targeting volunteer adult Internet-users aged 18 or older. Participants were included in the cohort after completing a baseline set of web questionnaires for collecting information on socio-demographic conditions, anthropometry, lifestyle, dietary intake (using repeated 24-h records) and physical activity along with health status, [22]. Baseline questionnaires were compared to traditional methods (paper forms or interview by a dietician) [23]–[25].

Approximately every month, they are invited to fill in optional complementary questionnaires related to determinants of food behavior and nutritional and health status.

### Ethics Statement

This study is being conducted according to guidelines laid down in the Declaration of Helsinki and was approved by the International Research Board of the French Institute for Health and Medical Research (IRB Inserm n° 0000388FWA00005831) and the “Comité National Informatique et Liberté” (CNIL n° 908450 and n° 909216). Electronic informed consent was obtained from all subjects.

### Data Collection

#### Organic food questionnaire

Two months after inclusion, participants were asked to provide information about organic products via an optional questionnaire. Questions were asked about opinions on prices, nutritional quality, taste and the health and environmental impact of organic products. Participants were also asked to report frequency of consumption/use, or else reasons for non-consumption/non-use of 18 organic products (fruit, vegetables, soya, dairy products, meat and fish, eggs, grains and legumes, bread and cereals, flour, vegetable oils and condiments, ready-to-eat meals, coffee/tea/herbal tea, wine, biscuits/chocolate/sugar/marmalade, other foods, dietary supplements, textiles and cosmetics). The eight possible responses were as follows: 1) most of the time; 2) occasionally; 3) never (too expensive); 4) never (product not available); 5) never (“I’m not interested in organic products”); 6) never (“I avoid such products”); 7) never (for no specific reason); and 8) “I don’t know”.

#### Socio-demographic and lifestyle data

At baseline, socio-demographic data included age, gender, education (≤ high school diploma, high school, post-secondary graduate), co-habitation or not, smoking status (never, former and current), number of children and income. Income per household unit was calculated using information about household income and composition. Thus, household income per month was divided by the number of consumption units (CU) calculated, i.e. 1 CU for the first adult in the household, 0.5 CU for other persons aged 14 or older and 0.3 CU for children under 14 [26]. The following categories of monthly income were used: <1,200, 1,200–1,800, 1,800–2,700 and >2,700 euros per household unit.

Leisure time physical activity was assessed using the French short form of the International Physical Activity Questionnaire (IPAQ), self-administered online [27]–[29]. Data obtained using IPAQ were computed for the metabolic equivalent task in min per week. The recommended IPAQ categories of physical activity were used: low (<30 min brisk walking/day), moderate (30–<60 min/day brisk walking/day or equivalent) and high (≥60 min brisk walking/day or equivalent).

The anthropometric questionnaire provided data on current height, weight and practice of restrictive diets (type and reason, history) [25].

#### Dietary data assessment

Dietary data were collected at baseline using three 24-h records randomly distributed within a two-week period, including two week days and one weekend day [22]. Participants reported all foods and beverages consumed throughout the day: breakfast, lunch, dinner and all other occasions. Portion sizes were then estimated using purchase unit, household unit and photographs, derived from a previously validated picture booklet [30]. No specific information was requested if foods eaten were organic or conventional. Consumption of fish and seafood per week was assessed by a specific frequency question. Nutrient intakes were estimated using the ad-hoc NutriNet-Santé composition table that includes more than 2,000 foods.

### Statistical Analysis and Data Treatment

Body mass index (BMI) was calculated as the ratio of weight in kilograms to squared height in meters (kg/m^2^).

In the present study, for each participant, daily mean food consumptions were calculated from 24-h records, weighting weekday or weekend to represent a week. Identification of underreporting participants was based on the validated published method proposed by Black [31] using Schofield equations for estimating resting metabolic rates [32].

For those with available data, we computed a score reflecting adherence to dietary components of the PNNS-GS (Programme National Nutrition Santé-Guidelines score) that reflects adherence to French nutritional recommendations [33], extensively described elsewhere [34]. Briefly, the original score includes 13 components: eight refer to food serving recommendations (fruit and vegetables, starchy foods, whole grain products, dairy products, meat, eggs and fish, seafood, vegetable fat, water and soda), four refer to moderation in consumption (added fat, salt, sweets, alcohol) and one represents physical activity. Points are deducted for overconsumption of salt and sweets and when energy intake exceeds the necessary energy level by more than 5%. Full details regarding the computation of this score can be found in **Table S1**. For the present analysis, we computed a modified version of the PNNS-GS (mPNNS-GS) which did not include the physical activity component.

We identified different profiles of attitude towards organic products. To do so, we used multiple correspondence analysis (MCA) [35] based on the 8 answer modalities to 18 questions concerning consumption/use of organic products. MCA enables extracting the dimensions that provide the most information on associations between responses.

The number of dimensions retained was determined according to the following criteria: eigenvalue >1, scree test and interpretability of extracted score. Then, cluster analysis was used to perform hierarchical ascendant classification using Ward’s method based on the first three dimensions retained in the MCA procedure [36].

To test the stability of the method, concordance between the classification performed on the whole sample and on a random sample including half of the population was tested. The kappa coefficient was high (85%). Besides, classification was stable across gender.

Due to well-known differences in dietary patterns between men and women, all subsequent analyses were stratified by gender.

In order to better understand the selected sample, we compared the characteristics of included and excluded NutriNet-Santé participants using chi-square tests and Student t-tests, as appropriate. Socio-demographic characteristics of the sample are presented in both men and women, as well as in the overall sample. For each individual, and to better describe clusters, we counted the number of times each of the 8 types of responses (i.e. most of the time, occasionally, never because too expensive, never because not available, never because not interested in organic products, never because “I avoid this product”, never (for no specific reason), “I don’t know”) was given to the 18 items. Profiles were described in terms of socio-demographic and lifestyle data, food group and nutrient intake by gender. P values referred to chi-square or non-parametric Kruskal-Wallis tests. Energy adjustment was performed using the residual method for nutrient intake. Univariate and multivariate models were performed to estimate the association between pre-overweight (excluding obesity) (25≤BMI<30) and obesity (BMI≥30) with profiles of organic food consumers using polytomous logistic regression (reference = BMI<25) [37]. Odds ratios and 95% confidence intervals were provided. The final model was adjusted for age, smoking status, physical activity, education, restrictive diet and quality of the diet (mPNNS-GS).

Tests of statistical significance were 2-sided and the type I error was set at 5%. Statistical analyses were performed using SAS software (version 9.1, SAS Institute Inc, Cary, NC, USA).

## Results

For the present analysis, we focused on participants included in the Nutrinet-Santé Study between June 2009 and December 2011. Among these 104,252 participants, we selected only those who filled in the second optional questionnaire (month 2) (N = 70,069), with complete and valid dietary data (three 24-h records) (N = 61,867), who were not underreporters (N = 54,322). We also eliminated those with missing covariates, leaving 54,311 participants in the present analysis.

Compared to excluded participants due to missing data, those included were older (43.7 versus 42.1 y), more often post-secondary graduate (64.5% versus 59.6%), showed a slightly lower BMI (23.8 versus 24.3 kg/m^2^), were more active (34.1% versus 33.8%) and less often current smokers (16.2% versus 20.2%) (**table S2**).

### Characteristics of the Sample

Descriptive information on the overall sample is presented in **Table 1**. Among the 54,311 participants, mean age was 43.7±14.4 and 77% were women, 64.5% had reached post-secondary degree and 49.8% were never-smokers. The average BMI was 23.8±4.5; 21.6% and 8.7% were overweight and obese, respectively.

**Table 1:** Characteristics of the NutriNet-Santé participants included in the present analysis; N = 54, 311^1^.

	Total	Men	Women	P^2^
N	54,311	12,405	41,906	
**Age (y)**	43.7 (14.4)	48.7 (15.1)	42.3 (13.9)	<.0001
**BMI (kg/m^2^)**	23.8 (4.5)	24.9 (3.9)	23.5 (4.6)	
**Education (%)**				
≤ High school diploma	18.7	23.8	17.2	<.0001
High school	16.8	13.7	17.8	
Post-secondary graduate	64.5	62.5	65.0	
**Monthly income per household unit** 3 **(%)**				<.0001
<1,200 euros	18.5	12.5	20.4	
1,200–1,800	27.9	25.6	28.7	
1,800–2,700	26.5	27.3	26.2	
>2,700	27.1	34.6	24.7	
**Tobacco use (%)**				<.0001
Never-smokers	49.8	42.2	52.1	
Former smokers	34.0	42.5	31.5	
Current smokers	16.2	15.3	16.4	

1 Values are means ± SD or % as appropriate.

2 P-values based on non-parametric Wilcoxon test or chi-squared test.

3 For 5,710 participants, these data were not available as the question was optional.

Organic products were perceived as being better for health and the environment by 69.9% and 83.7% of the participants, respectively. However, 51% non-consumers declared that they were too expensive (**table S3**).

### Profiles of Organic Product Consumers

We identified 5 clusters (clusters 1 to 5) as shown in **Table 2**. Two of these were composed of consumers of organic products (COP), including regular consumers (cluster 5: RCOP) and occasional consumers (cluster 4: OCOP). Most participants were occasional consumers (OCOP); 52% were women and 48% men. Moreover, RCOP comprised 11% men and 15% women, respectively. Three other clusters grouped individuals who generally did not consume organic products due to the high cost (cluster 3), because they avoided such products (cluster 2) or because they were not interested in organic products (cluster 1).

**Table 2:** Types of responses to the 18 items concerning frequency of organic product consumption across clusters, NutriNet-Santé Study, N = 54, 311^1,2^.

	Cluster 1	Cluster 2	Cluster 3	Cluster 4	Cluster 5
	Not interested	Avoidance	Too expensive	OCOP	RCOP
**N (%)**	9,009 (16.6)	5,700 (10.5)	4,484 (8.3)	27,512 (50.7)	7,606 (14.0)
**Men: N (%)**	2,843 (22.9)	1,423(11.5)	840 (6.8)	5,925 (47.8)	1,374 (11.1)
**Women: N (%)**	6,166 (14.7)	4,277 (10.2)	3,644 (8.7)	21,587 (51.5)	6,232 (14.9)
**Most of the time**	0.18 (0.47)	0.18 (0.52)	0.14 (0.41)	1.25 (1.54)	**8.51 (2.95)**
**Occasionally**	2.57 (2.26)	1.79 (2.06)	1.72 (1.81)	**7.28 (3.41)**	**6.02 (2.88)**
**Never; too expensive**	1.55 (1.32)	1.4 (1.42)	**12.97 (2.47)**	2.65 (2.72)	0.41 (0.69)
**Never; not available**	0.28 (0.7)	0.51 (0.74)	0.35 (0.83)	0.97 (1.7)	0.48 (0.85)
**Never; I’m not interested in organic products**	**10.85 (3.19)**	1.57 (2.23)	0.55 (0.89)	2.14 (2.09)	0.39 (0.72)
**Never; I avoid organic products**	0.59 (1.06)	**2.20 (4.22)**	0.38 (0.79)	0.59 (1.04)	0.46 (0.75)
**Never (no specific reason)**	1.54 (1.38)	**9.51 (4.24)**	1.69 (1.41)	2.24 (1.67)	1.44 (1.24)
**I don’t know**	0.45 (1.21)	0.84 (1.44)	0.19 (0.58)	0.89 (1.74)	0.30 (0.58)

OCOP: occasional consumers of organic products, RCOP: regular consumers of organic products.

1 Values are means (SD) of the number of occurrences of each type of response to the 18 questions: fruit, vegetables, soya, dairy products, meat and fish, eggs, grains and legumes, bread and cereals, flour, vegetable oil and condiments, ready-to-eat meals, coffee/tea/herbal tea, wine, biscuits/chocolate/sugar/marmalade, other foods, dietary supplements, textiles, cosmetics. Total: 18.

2 Clusters were identified using MCA based on the 18 items questioning attitudes towards organic products. Next, cluster analysis was used to perform hierarchical ascendant classification using Ward’s method based on the first three dimensions retained from the MCA procedure.

General characteristics across clusters and genders are presented in **Table 3**. RCOP males were younger and more often never-smokers than others, while RCOP females were older and more often former smokers. In both genders, consumption of organic foods was associated with a higher education level, lower BMI and higher level of physical activity along with less frequent restrictive dieting. As expected, cluster 3 participants, i.e. those who stated that organic foods were too expensive, had a lower income and education level. They also more often reported a restrictive diet. Income per household unit in the other four clusters was high and fairly similar among clusters. In addition, participants who were uninterested in organic products (cluster 1) displayed weaker adherence to nutritional guidelines compared to RCOP (**Table 3**): 7.7±1.7 versus 8.4±1.8 in men, respectively and 7.9±1.8 versus 8.7±1.7 in women, respectively. Adherence to nutritional guidelines was similar in clusters 1, 2 and 3.

**Table 3:** Descriptive characteristics of organic consumption clusters by gender, NutriNet-Santé Study, N = 54,311^1^.

	Men						Women					
	Cluster 1	Cluster 2	Cluster 3	Cluster 4	Cluster 5	P^2^	Cluster 1	Cluster 2	Cluster 3	Cluster 4	Cluster 5	P^2^
	Not interested	Avoidance	Too expensive	OCOP	RCOP		Not interested	Avoidance	Too expensive	OCOP	RCOP	
**N**	2,843	1,423	840	5,925	1,374		6,166	4,277	3,644	21,587	6,232	
**Age (y)** 3	48.8 (15.7)^a^	51.1 (16.0)^b^	49.6 (14.4)^a,b^	48.3 (14.8)^a,c^	47.1 (13.8)^c^	<.0001	40.4 (14.5)^a^	42.3 (14.3)^b^	40.4 (13.4)^a^	42.3 (13.9)^c^	45.1 (13.0)^d^	<.0001
**mPNNS-GS** 3	7.7 (1.7)^a^	7.6 (1.8)^a^	7.7 (1.8)^a^	8.0 (1.7)^b^	8.4 (1.8)^ b^	<.0001	7.9 (1.8)^a^	7.8 (1.8)^a,b^	7.7 (1.8)^b^	8.2 (1.8)^c^	8.7 (1.7)^d^	<.0001
**BMI (kg/m^2^)** 3	25.2 (3.9)^a^	25.5 (4.1)^a^	25.5 (4.1)^a^	24.8 (3.8)^b^	23.7 (3.2)^c^	<.0001	23.6 (4.6)^a^	24.1 (5.1)^b^	24.2 (5.2)^b^	23.4 (4.5)^a^	22.6 (3.9)^c^	<.0001
**Restrictive diet (%)**	24.6	25.9	29.4	25.3	22.1	<.0001	52.9	54.4	59.1	54.2	51.1	<.0001
**Education (%)**						<.0001						<.0001
≤ High school diploma	22.1	30.6	36.3	22.9	16.5		15.1	24.5	24.3	16.3	13.5	
Trade/certificate	13.9	14.9	16.7	13.2	12.2		18.5	21.4	22.3	17.1	14.1	
Post-secondary degree	63.9	54.5	47.0	63.9	71.3		66.4	54.1	53.3	66.7	72.4	
**Monthly income per household unit**						<.0001						<.0001
<1,200 euros	10.8	14.3	23.1	11.8	11.2		18.0	25.2	33.3	19.5	15.0	
1,200–1,800	23.6	27.4	33.6	25.2	24.8		27.2	29.4	34.9	28.5	26.8	
1,800–2,700	27.2	25.4	26.2	27.9	27.1		26.9	23.3	22.0	26.8	28.0	
>2,700	38.4	33.0	17.2	35.1	36.9		27.8	22.1	9.7	25.3	30.1	
**Tobacco use (%)**						0.46						<.0001
Never-smokers	42.2	39.6	42.0	42.4	44.4		54.2	54.1	50.9	51.9	49.7	
Former smokers	42.1	45.0	42.5	42.3	41.1		28.2	29.2	30.8	31.5	36.9	
Current smokers	15.7	15.3	15.5	15.2	14.5		17.6	16.6	18.3	16.6	13.4	
**Physical activity (%)**						<.0001						<.0001
Low	19.0	18.3	20.4	16.6	14.0		21.7	20.0	23.0	17.7	15.8	
Medium	30.7	26.6	29.4	30.3	34.9		34.6	29.4	31.3	35.5	36.2	
High	34.3	36.2	33.6	35.8	37.6		21.3	24.2	22.5	24.4	28.2	

1 Values are means (SD) or % as appropriate.

2 P-value is based on the non-parametric Kruskal-Wallis test for heterogeneity between groups or chi-square test.

3 Means annotated with the same letter are not different (P<0.05), Tukey post-hoc test.

### Dietary Intake According to Profile of Organic Product Consumers

Food intakes for the different clusters are shown in **Table 4**. For clarity, we focused on differences greater than 20%. Compared to RCOP participants, those in cluster 1 showed lower consumption of healthy foods such as fruit (−20% in men, −31% in women), vegetables (−27% in men, −28% in women), legumes (−49% in men, −85% in women), vegetable oils (−38% in men, +36% in women), whole grains (−247% in men, −153% in women) and nuts (−239% in men, −381% in women) and higher consumption of sweet soft drinks (+34% in men, +46% in women) and alcoholic beverages (+18% in men, +8% in women), animal products including processed meat (+31% in both genders) and fresh meat (+34% in men, +32% in women) and milk (+43% in both genders). Participants in clusters 2 and 3 showed overall comparable differences in dietary patterns to those of cluster 1 with respect to RCOP. It is noteworthy that OCOP consumers (cluster 4) of organic foods showed profiles intermediate between never-consumers and RCOP.

**Table 4:** Food consumption (g/d) across organic consumption clusters by gender, NutriNet-Santé Study, N = 54, 311^1^.

	Men						Women					
	Cluster 1	Cluster 2	Cluster 3	Cluster 4	Cluster 5	P^2^	Cluster 1	Cluster 2	Cluster 3	Cluster 4	Cluster 5	P^2^
	Not interested	Avoidance	Too expensive	OCOP	RCOP		Not interested	Avoidance	Too expensive	OCOP	RCOP	
Vegetables	186 (181–190)	180 (174–187)	185 (176–193)	203 (200–207)	236 (229–243)	<0.0001	179 (176–181)	173 (170–176)	180 (176–183)	195 (194–197)	228 (226–231)	<0.0001
Fruit	181 (175–187)	178 (170–187)	172 (162–183)	193 (189–197)	218 (209–226)	<0.0001	160 (157–164)	149 (145–154)	153 (149–158)	175 (174–177)	210 (206–213)	<0.0001
Dried fruit	1.8 (1.5–2.1)	1.5 (1.1–1.9)	1.6 (1.0–2.2)	2.1 (1.8–2.3)	4.9 (4.4–5.3)	<0.0001	1.2 (1.1–1.4)	1.1 (0.9–1.3)	1.2 (1.0–1.4)	1.8 (1.7–1.9)	3.6 (3.5–3.8)	<0.0001
Fruit juice	61 (58–65)	52 (47–56)	53 (46–59)	65 (62–67)	67 (62–72)	<0.0001	57 (55–59)	50 (48–53)	50 (47–53)	54 (53––55)	52 (51–54)	<0.0001
Vegetable juice	1.1 (0.5–1.7)	1.8 (0.9–2.6)	0.9 (−0.2–2.0)	1.7 (1.3–2.1)	3.3 (2.4–4.2)	<0.0001	1.1 (0.8–1.5)	1.1 (0.7–1.5)	1.3 (0.9–1.8)	1.5 (1.3–1.7)	3.5 (3.1–3.8)	<0.0001
Nuts	2.5 (2.0–3.0)	2.6 (1.9–3.3)	2.4 (1.5–3.2)	3.0 (2.7–3.3)	8.5 (7.8–9.2)	<0.0001	1.5 (1.2–1.7)	1.3 (1.0–1.5)	1.5 (1.1–1.8)	2.7 (2.5–2.8)	7.2 (6.9–7.4)	<0.0001
Legumes	9.4 (8.4–10.4)	8.1 (6.7–9.4)	11 (9–13)	9.7 (9.0–10.3)	14 (13–15)	<0.0001	6.4 (5.9–7.0)	6.0 (5.4–6.6)	7.3 (6.6–8.0)	7.8 (7.5–8.1)	12 (11–12)	<0.0001
Potatoes and other tubers	53 (50–55)	56 (53–59)	54 (50–58)	52 (50–53)	53 (50–56)	0.15	39 (38–41)	41 (39–42)	41 (40–43)	38 (37–39)	39 (38–40)	<0.0001
Soup	47 (44–51)	51 (46–56)	52 (45–59)	55 (53–58)	64 (58–69)	<0.0001	42 (40–44)	43 (41–46)	43 (40–46)	51 (49–52)	62 (60–64)	<0.0001
Whole grains	14 (13–16)	13 (11–15)	15 (12–18)	21 (20–22)	49 (47–52)	<0.0001	13 (12–14)	12 (11–13)	13 (12–15)	17 (17–18)	33 (32–34)	<0.0001
Refined cereals	188 (185–192)	178 (173–184)	188 (181–194)	190 (188–193)	185 (179–190)	<0.0001	140 (138–142)	137 (135–139)	141 (139–144)	142 (141–143)	137 (136–139)	<0.0001
Fish and seafood	46 (44–48)	45 (42–48)	42 (39–46)	47 (46–49)	47 (45–50)	0.01	39 (38–40)	37 (35–38)	36 (34–37)	41 (40–41)	41 (40–42)	<0.0001
Meat	58 (56–60)	59 (56–61)	59 (56–62)	51 (50–53)	38 (35–41)	<0.0001	42 (41–43)	44 (43–45)	41 (40–42)	38 (37–38)	29 (28–30)	<0.0001
Poultry	29 (28–31)	30 (28–32)	28 (26–31)	30 (29–30)	22 (20–24)	<0.0001	25 (24–25)	25 (24–26)	24 (23–25)	24 (23–24)	19 (19–20)	<0.0001
Offal	5.7 (5.1–6.4)	5.2 (4.3–6.1)	5.4 (4.3–6.6)	5.2 (4.7–5.6)	4.1 (3.2–4.9)	0.03	2.9 (2.6–3.2)	3.4 (3.1–3.8)	3.1 (2.7–3.5)	3.0 (2.8–3.2)	2.6 (2.3–2.9)	0.04
Ham	11 (10–11)	12 (11–13)	13 (11–14)	10 (10–11)	7.8 (6.8–8.8)	<0.0001	10 (10–11)	12 (11–12)	12 (11–12)	9.9 (9.7–10.2)	7.0 (6.6–7.4)	<0.0001
Processed meat	30 (29–32)	31 (29–33)	33 (31–35)	26 (26–27)	21 (19–23)	<0.0001	19 (19–20)	20 (19–20)	20 (19–21)	17 (17–17)	13 (13–14)	<0.0001
Egg	13 (12–14)	14 (12–15)	14 (13–16)	13 (13–14)	16 (14–17)	0.002	11 (11–12)	12 (11–12)	11 (11–12)	12 (12–12)	13 (13–14)	<0.0001
Oil	8.1 (7.8–8.4)	7.6 (7.2–8.1)	8.1 (7.5–8.7)	8.5 (8.3–8.8)	11 (11–12)	<0.0001	6.9 (6.8–7.1)	6.7 (6.4–6.9)	6.6 (6.4–6.8)	7.4 (7.3–7.5)	9.5 (9.3–9.6)	<0.0001
Butter	7.1 (6.8–7.5)	7.7 (7.3–8.2)	6.7 (6.1–7.3)	7.0 (6.7–7.2)	6.9 (6.4–7.3)	0.04	6.1 (5.9–6.3)	6.2 (6.0–6.4)	6.2 (6.0–6.4)	6.1 (6.0–6.2)	5.9 (5.8–6.1)	0.01
Margarines	2.5 (2.2–2.7)	2.6 (2.2–2.9)	3.4 (3.0–3.9)	2.4 (2.3–2.6)	1.7 (1.3–2.0)	<0.0001	1.6 (1.5–1.7)	2.0 (1.8–2.1)	2.1 (1.9–2.2)	1.7 (1.6–1.8)	1.3 (1.2–1.4)	<0.0001
Other added fats	3.4 (3.1–3.7)	3.5 (3.0–3.9)	3.6 (3.0–4.1)	3.4 (3.2–3.6)	3.2 (2.8–3.6)	0.35	4.3 (4.1–4.5)	4.0 (3.8–4.3)	4.4 (4.2–4.7)	4.0 (3.9–4.1)	3.5 (3.3–3.7)	0.001
Dressing	19 (19–20)	18 (17–19)	18 (16–19)	17 (17–18)	15 (14–16)	<0.0001	15 (15–16)	15 (14–15)	15 (15–16)	14 (14–14)	12 (12–13)	<0.0001
Milk	103 (98–108)	99 (92–106)	110 (101–119)	90 (86–93)	59 (52–66)	<0.0001	87 (84–90)	89 (85–92)	102 (98–106)	79 (77–80)	50 (47–53)	<0.0001
Cheese	40 (39–42)	38 (37–40)	41 (39–43)	41 (40–42)	43 (42–45)	0.02	30 (29–30)	29 (29–30)	29 (28–30)	31 (30–31)	33 (32–34)	<0.0001
Dairy products^3^	69 (66–71)	72 (68–75)	74 (69–79)	71 (69–73)	60 (56–64)	<0.0001	78 (76–80)	81 (78–83)	80 (78–83)	81 (80–82)	69 (67–71)	<0.0001
Milky dessert	38 (36–40)	36 (33–39)	39 (35–43)	37 (36–38)	37 (34–40)	0.83	34 (33–36)	36 (35–38)	38 (36–39)	34 (34–35)	35 (34–36)	<0.0001
Cookies, biscuits^4^	62 (60–65)	61 (57–64)	61 (56–65)	61 (59–63)	63 (59–66)	0.27	60 (58–61)	54 (53–56)	56 (54–58)	56 (55–57)	53 (51–54)	<0.0001
Soft drinks	64 (60–68)	59 (53–66)	63 (55–71)	53 (50–56)	42 (36–49)	0.0002	56 (54–59)	56 (53–59)	57 (54–61)	42 (41–44)	30 (28–33)	<0.0001
Alcoholic beverages	199 (191–207)	190 (179–201)	157 (143–172)	182 (176–187)	164 (152–175)	<0.0001	79 (76–82)	71 (68–75)	61 (57–65)	74 (73–76)	73 (70–76)	<0.0001
Sweet products^5^	29 (28–30)	28 (26–29)	26 (24–29)	31 (30–32)	35 (33–36)	<0.0001	24 (23–25)	24 (23–24)	24 (23–25)	25 (25–26)	26 (26–27)	<0.0001
Sweet and fat products^6^	23 (22–25)	24 (22–26)	22 (20–24)	22 (22–23)	19 (17–21)	0.22	23 (23–24)	22 (21–23)	23 (22–24)	21 (20–21)	18 (17–18)	<0.0001
Fast food^7^	41 (39–44)	39 (36–42)	37 (33–41)	38 (36–39)	32 (29–35)	<0.0001	36 (35–37)	34 (33–36)	36 (34–37)	32 (31–32)	27 (25–28)	<0.0001

1 Values are means (95% confidence interval).

2 P-value is based on the non-parametric Kruskal-Wallis test for heterogeneity between groups.

3 Yogurt, cottage cheese.

4 Including croissants and pastries.

5 Candy, confectionery, honey, jam, all types of sugars, coulis, syrup, sorbet.

6 Chocolates, chocolate bars, ice cream, ice cream bars, chocolate paste, almond paste.

7 Pizzas, burgers, quiches, salted pies, donuts, savory samosas, salted cakes, salted pastries, hot dog, cheese nans, stuffed pancakes and croque monsieur, nems, etc.

Differences in energy intake and in other macronutrients across clusters were low (**Table 5**). Compared to RCOP, participants in cluster 1 had lower intakes of polyunsaturated fatty acids (−12% in both genders), especially n-3 PUFA (−19% in men, −20% in women), fibers (−27% in men, −28% in women), beta-carotene (−28% in men, −33% in women), folic acid (−15% in men, −17% in women), vitamin C (−10% in men, −13% in women) and iron (−20% in men, −18% in women). They were also characterized by a higher alcohol intake (+17% in men, +11% in women) and cholesterol (+12% in men, +10% in women). As was the case for food consumption, differences in nutrient intakes of cluster 2 and cluster 3 participants were generally comparable to those of cluster 1 with respect to RCOP, while OCOP (cluster 4) showed intermediate profiles.

**Table 5:** Daily nutrient intake across organic consumption clusters by gender, NutriNet-Santé Study, N = 54, 311^1^.

	Men						Women					
	Cluster 1	Cluster 2	Cluster 3	Cluster 4	Cluster 5	P^2^	Cluster 1	Cluster 2	Cluster 3	Cluster 4	Cluster 5	P^2^
	Not interested	Avoidance	Too expensive	OCOP	RCOP		Not interested	Avoidance	Too expensive	OCOP	RCOP	
Energy (Kcal/d)	2214 (2194–2233)	2156 (2129–2183)	2166 (2130–2201)	2182 (2169–2195)	2204 (2176–2231)	0.01	1757 (1747–1768)	1714 (1702–1727)	1742 (1728–1755)	1739 (1734–1745)	1741 (1731–1752)	<0.0001
Carbohydrates^3^ (%)	37.3 (37.0–37.5)	36.9 (36.6–37.3)	37.3 (36.8–37.8)	38.2 (38.0–38.3)	39.5 (39.1–39.8)	<0.0001	38.4 (38.2–38.5)	38.2 (38.0–38.4)	38.9 (38.7–39.1)	38.8 (38.7–38.9)	39.4 (39.3–39.6)	<0.0001
Lipids^3^ (%)	37.8 (37.5–38.0)	37.7 (37.4–38.1)	38.1 (37.6–38.5)	37.3 (37.1–37.4)	37.5 (37.1–37.8)	0.0003	38.7 (38.6–38.9)	38.7 (38.5–38.8)	38.6 (38.4–38.8)	38.4 (38.3–38.5)	38.8 (38.6–38.9)	<0.0001
Proteins^3^ (%)	17.1 (17.0–17.3)	17.4 (17.3–17.6)	17.6 (17.4–17.9)	17.1 (17.0–17.2)	15.9 (15.7–16.1)	<0.0001	17.4 (17.3–17.5)	17.9 (17.8–18.0)	17.6 (17.4–17.7)	17.4 (17.4–17.5)	16.5 (16.4–16.6)	<0.0001
Alcohol (%)	16.3 (15.7–17.0)	16.2 (15.3–17.1)	13.5 (12.3–14.7)	14.9 (14.4–15.3)	13.5 (12.6–14.5)	<0.0001	6.7 (6.4–6.9)	6.1 (5.8–6.4)	5.2 (4.9–5.5)	6.2 (6.1–6.3)	6.0 (5.7–6.2)	<0.0001
Saturated fatty acids^3^ (%)	15.6 (15.4–15.7)	15.7 (15.5–15.9)	15.6 (15.3–15.8)	15.2 (15.1–15.3)	14.5 (14.3–14.7)	<0.0001	16.1 (16.0–16.1)	16.1 (16.0–16.2)	16.0 (15.9–16.2)	15.7 (15.7–15.8)	15.0 (14.9–15.1)	<0.0001
Monounsaturated fatty acids^3^ (%)	14.1 (13.9–14.2)	13.9 (13.7–14.0)	14.2 (14.0–14.4)	13.9 (13.8–14.0)	14.3 (14.1–14.5)	0.002	14.4 (14.3–14.5)	14.3 (14.2–14.4)	14.3 (14.2–14.4)	14.3 (14.3–14.4)	14.9 (14.8–14.9)	<0.0001
Polyunsaturated fatty acids^3^ (%)	5.3 (5.2–5.4)	5.3 (5.2–5.4)	5.4 (5.3–5.6)	5.3 (5.3–5.4)	5.9 (5.8–6.1)	<0.0001	5.4 (5.3–5.4)	5.4 (5.3–5.4)	5.4 (5.4–5.5)	5.5 (5.4–5.5)	6.0 (6.0–6.1)	<0.0001
N-6 Polyunsaturated fatty acids^4^ (g/d)	10.7 (10.6–10.9)	10.8 (10.6–11.1)	10.9 (10.6–11.2)	10.6 (10.5–10.8)	11.9 (11.7–12.2)	<0.0001	8.5 (8.4–8.6)	8.6 (8.5–8.7)	8.7 (8.6–8.9)	8.7 (8.6–8.7)	9.6 (9.5–9.7)	<0.0001
N-3 Polyunsaturated fatty acids^4^ (g/d)	1.5 (1.4–1.5)	1.5 (1.4–1.5)	1.5 (1.4–1.6)	1.6 (1.5–1.6)	1.8 (1.7–1.8)	<0.0001	1.2 (1.2–1.2)	1.2 (1.2–1.2)	1.2 (1.2–1.2)	1.3 (1.3–1.3)	1.5 (1.5–1.5)	<0.0001
Eicosapentaenoic acid 4(g/d)	0.14 (0.14–0.15)	0.14 (0.13–0.15)	0.13 (0.12–0.15)	0.15 (0.14–0.15)	0.16 (0.15–0.17)	0.002	0.12 (0.11–0.12)	0.11 (0.10–0.11)	0.10 (0.10–0.11)	0.12 (0.12–0.12)	0.13 (0.13–0.14)	<0.0001
Docosaheaxaenoic acid 4(g/d)	0.22 (0.21–0.23)	0.21 (0.19–0.22)	0.21 (0.19–0.23)	0.23 (0.22–0.24)	0.24 (0.23–0.26)	0.003	0.18 (0.17–0.19)	0.16 (0.16–0.17)	0.16 (0.15–0.17)	0.19 (0.18–0.19)	0.20 (0.19–0.21)	<0.0001
Cholesterol^4^ (mg/d)	386 (381–391)	391 (384–398)	387 (378–397)	376 (372–380)	340 (332–347)	<0.0001	313 (310–315)	319 (316–323)	308 (305–312)	306 (304–307)	281 (279–284)	<0.0001
Beta-carotene^4^ (µg/d)	3285 (3180–3391)	3190 (3042–3339)	3336 (3143–3529)	3592 (3519–3665)	4217 (4066–4368)	<0.0001	3080 (3017–3143)	2950 (2874–3026)	3076 (2994–3159)	3361 (3327–3394)	4083 (4020–4145)	<0.0001
Calcium^4^ (mg/d)	966 (955–976)	960 (945–974)	986 (967–1005)	983 (975–990)	962 (947–977)	0.01	838 (832–844)	845 (838–852)	849 (841–857)	852 (848–855)	837 (830–843)	<0.0001
Iron^4^ (mg/d)	14.6 (14.4–14.8)	14.4 (14.2–14.7)	14.6 (14.3–14.9)	15.3 (15.2–15.5)	17.5 (17.3–17.7)	<0.0001	11.8 (11.7–11.9)	11.6 (11.5–11.7)	11.8 (11.7–11.9)	12.5 (12.4–12.5)	13.9 (13.8–14.0)	<0.0001
Potassium^4^ (mg/d)	3244 (3219–3269)	3241 (3205–3277)	3246 (3200–3293)	3314 (3296–3332)	3418 (3382–3455)	<0.0001	2738 (2722–2754)	2716 (2697–2735)	2753 (2732–2773)	2818 (2810–2827)	2945 (2929–2960)	<0.0001
Magnesium^4^ (mg/d)	350 (346–353)	345 (340–350)	344 (337–350)	369 (367–371)	413 (408–418)	<0.0001	291 (289–293)	284 (282–287)	288 (286–291)	308 (307–309)	345 (343–347)	<0.0001
Sodium4 (mg/d)	3017 (2991–3044)	3046 (3008–3083)	3033 (2984–3082)	2993 (2974–3011)	2837 (2799–2876)	<0.0001	2364 (2349–2378)	2377 (2359–2395)	2375 (2356–2394)	2346 (2339–2354)	2240 (2225–2254)	<0.0001
Phosphorus^4^ (mg/d)	1387 (1378–1397)	1390 (1376–1404)	1407 (1389–1425)	1403 (1397–1410)	1406 (1392–1419)	0.06	1160 (1155–1166)	1168 (1161–1176)	1169 (1161–1177)	1175 (1172–1179)	1173 (1167–1179)	0.002
Retinol^4^ (mg/d)	608 (577–639)	628 (584–672)	593 (536–650)	596 (575–618)	538 (494–583)	<0.0001	468 (451–486)	488 (467–509)	463 (440–485)	467 (457–476)	454 (437–471)	<0.0001
Vitamin B1^4^ (mg/d)	1.3 (1.3–1.4)	1.4 (1.3–1.4)	1.4 (1.4–1.5)	1.4 (1.4–1.4)	1.4 (1.4–1.5)	0.004	1.1 (1.1–1.2)	1.1 (1.1–1.2)	1.2 (1.2–1.2)	1.2 (1.2–1.2)	1.2 (1.2–1.2)	<0.0001
Vitamin B2^4^ (mg/d)	1.9 (1.9–1.9)	1.9 (1.9–1.9)	1.9 (1.9–2.0)	1.9 (1.9–1.9)	1.9 (1.9–1.9)	0.001	1.6 (1.6–1.6)	1.6 (1.6–1.7)	1.7 (1.6–1.7)	1.7 (1.6–1.7)	1.6 (1.6–1.6)	<0.0001
Vitamin B3^4^ (mg/d)	21.3 (21.1–21.5)	21.4 (21.1–21.7)	21.1 (20.6–21.5)	21.6 (21.4–21.8)	21.3 (21.0–21.6)	0.11	17.5 (17.3–17.6)	17.2 (17.1–17.4)	17.1 (17.0–17.3)	17.5 (17.4–17.6)	17.4 (17.3–17.5)	0.0003
Vitamin B5^4^ (mg/d)	5.9 (5.9–6.0)	6.0 (5.9–6.0)	6.0 (5.9–6.1)	6.0 (6.0–6.0)	5.9 (5.8–6.0)	0.03	4.9 (4.9–5.0)	4.9 (4.9–5.0)	5.0 (4.9–5.0)	5.0 (5.0––5.0)	5.0 (4.9–5.0)	0.01
Vitamin B6^4^ (mg/d)	1.9 (1.9–2.0)	1.9 (1.9–2.0)	1.9 (1.9–2.0)	2.0 (2.0–2.0)	2.1 (2.1–2.2)	<0.0001	1.6 (1.6–1.6)	1.6 (1.6–1.6)	1.6 (1.6–1.6)	1.6 (1.6–1.6)	1.7 (1.7–1.8)	<0.0001
Vitamin B9^4^ (µg/d)	345 (341–349)	342 (337–348)	343 (335–351)	363 (360–366)	397 (391–403)	<0.0001	304 (302–307)	297 (294–300)	301 (298–305)	321 (319–322)	355 (353–358)	<0.0001
Vitamin B12^4^ (mg/d)	6.6 (6.3–6.8)	6.7 (6.4–7.1)	6.4 (5.9–6.8)	6.6 (6.4–6.7)	5.9 (5.6–6.2)	<0.0001	4.9 (4.8–5.1)	5.1 (4.9–5.2)	4.8 (4.7–5.0)	5.0 (4.9–5.0)	4.7 (4.6–4.9)	<0.0001
Vitamin C^4^ (mg/d)	114 (112–117)	112 (108–116)	113 (108–118)	121 (119–122)	126 (122–129)	<0.0001	106 (105–108)	102 (99–104)	104 (101–106)	112 (111–113)	120 (118–122)	<0.0001
Vitamin D^4^ (µg/d)	3.1 (3.0–3.2)	3.0 (2.9–3.2)	3.1 (2.9–3.3)	3.2 (3.1–3.3)	3.2 (3.1–3.4)	0.68	2.6 (2.5–2.6)	2.5 (2.4–2.6)	2.5 (2.4–2.6)	2.6 (2.6–2.7)	2.7 (2.7–2.8)	0.002
Vitamin E^4^ (mg/d)	12.5 (12.3–12.6)	12.1 (11.9–12.4)	12.7 (12.4–13.0)	12.5 (12.3–12.6)	13.6 (13.3–13.8)	<0.0001	10.6 (10.5–10.7)	10.6 (10.4–10.7)	10.8 (10.7–10.9)	10.8 (10.8–10.9)	11.8 (11.7–11.9)	<0.0001
Fiber^4^ (g/d)	19.8 (19.5–20.0)	19.5 (19.2–19.9)	19.8 (19.3–20.3)	21.3 (21.1–21.5)	25.1 (24.8–25.5)	<0.0001	16.8 (16.6–16.9)	16.3 (16.1–16.5)	16.9 (16.7–17.0)	18.2 (18.1–18.3)	21.5 (21.3–21.6)	<0.0001

1 Values are means (95% confidence interval).

2 P value is based on the non-parametric Kruskal-Wallis test for heterogeneity between groups.

3 Expressed as percent of energy.

4 Energy-adjusted using the residual method.

### Association between BMI Categories and Profiles of Organic Product Consumers

The association between overweight/obesity and profiles of organic food consumers are presented in **Table 6**. In the unadjusted model, among men and women, participants in the RCOP (cluster 5) group had a significantly lower probability of being overweight and obese than those who did not eat organic food (cluster 1). OCOP displayed intermediate figures. Compared with cluster 1, persons who avoided organic products (cluster 2) were more likely to be overweight (in both gender) or obese (in women only) and those who did not buy any organic food due to high cost (cluster 3) were more likely to be obese. After adjustment for age, physical activity, education, smoking, energy intake, use of restrictive diet and the PNNS dietary adequacy score, RCOP in cluster 5 conserved a markedly lower probability of being overweight or obese: −36% and −62% in men and −42% and −48% in women, respectively. For OCOP (cluster 4), women showed a 12% and 13% lower probability of being overweight or obese, respectively, whereas men no longer showed a reduced risk after adjustments. Women who avoided or did not buy organic food because of the high cost showed greater probability of being obese than those in cluster 1.

**Table 6:** Association between organic food consumption cluster and BMI categories by gender, NutriNet-Santé Study, N = 54, 311^1^.

	Men					Women				
	Cluster 1	Cluster 2	Cluster 3	Cluster 4	Cluster 5	Cluster 1	Cluster 2	Cluster 3	Cluster 4	Cluster 5
	Not interested	Avoidance	Too expensive	OCOP	RCOP	Not interested	Avoidance	Too expensive	OCOP	RCOP
**Overweight**										
Prevalence	34.6%	39.9	35.6%	32.5%	25.3%	19.0%	21.0%	19.7%	18.3%	13.6%
Model a^2^	1 Ref.	**1.29 (1.13–1.48)**	1.10 (0.93–1.30)	**0.89 (0.80–0.98)**	**0.57 (0.50–0.66)**	1 Ref.	**1.18 (1.07–1.30)**	1.10 (0.99–1.22)	0.94 (0.88–1.01)	**0.64 (0.58–0.70)**
Model b^3^	1 Ref.	**1.19 (1.03–1.37)**	0.99 (0.83–1.18)	**0.88 (0.79–0.97)**	**0.58 (0.50–0.68)**	1 Ref.	**1.11 (1.00–1.23)**	0.99 (0.89–1.11)	**0.89 (0.82–0.96)**	**0.57 (0.52–0.63)**
Model c^4^	1 Ref.	1.15 (0.97–1.35)	0.95 (0.78–1.17)	0.93 (0.83–1.04)	**0.64 (0.53–0.76)**	1 Ref.	1.05 (0.93–1.19)	0.95 (0.83–1.08)	**0.88 (0.80–0.96)**	**0.58 (0.52–0.65)**
**Obesity**										
Prevalence	10.1%	10.5%	12.5%	8.7%	4.3%	9.0%	11.7%	12.8%	8.2%	5.3%
Model a^2^	1 Ref.	1.17 (0.94–1.45)	**1.32 (1.03–1.69)**	**0.81 (0.69–0.95)**	**0.33 (0.25–0.45)**	1 Ref.	**1.40 (1.23–1.59)**	**1.52 (1.33–1.74)**	**0.90 (0.81–1.00)**	**0.52 (0.45–0.60)**
Model b^3^	1 Ref.	1.05 (0.83–1.34)	1.04 (0.79–1.37)	**0.79 (0.67–0.94)**	**0.34 (0.25–0.46)**	1 Ref.	**1.31 (1.14–1.51)**	**1.27 (1.10–1.47)**	**0.86 (0.77–0.96)**	**0.49 (0.42–0.57)**
Model c^4^	1 Ref.	1.04 (0.79–1.36)	1.10 (0.81–1.51)	0.89 (0.73–1.09)	**0.38 (0.27–0.55)**	1 Ref.	**1.33 (1.13–1.57)**	**1.24 (1.04–1.47)**	**0.87 (0.77–0.99)**	**0.52 (0.43–0.61)**

1 Values are odds ratios (95% confidence interval) estimated through polytomous logistic regression using BMI<25 as a reference, all P-values for Wald test of the global effect between clusters <0.0001.

2 Model a is unadjusted.

3 Model b: model a+age, physical activity, education, smoking, energy intake, restrictive diet.

4 Model c: model b+mPNNS-GS; due to missing value for mPNNS-GS, numbers of participants across clusters were 2,248, 1,106, 669, 4,603 and 1,080 in men and 4,727, 3,112, 2,719, 16,381 and4,891 in women (Cluster 1 to Cluster 5).

## Discussion

The present study is the first to describe, for a large cohort, socio-demographic aspects, lifestyle and dietary patterns of adult consumers of organic foods compared to non-consumers. We identified 5 typical clusters of consumers based on their attitude towards organic foods, including two that comprised occasional and regular organic food consumers. Compared to the 3 clusters of non-consumers, organic food consumers progressively improved adherence to the recommended food pattern and nutrient intake and had lower probability of being overweight or obese, after accounting for confounding factors.

### Profiles and Attitudes of Organic Product Consumers

Based on the frequency of organic product consumption, three clusters grouped together non-consumers of organic foods, mainly because they were either uninterested in these products, deliberately avoided them or considered them too expensive. In contrast, two other clusters grouped occasional and regular consumers of organic products. The present findings support previous research showing that, in France, most organic product purchases are occasional; indeed, only 6% of the general population reported daily organic product purchases [13]. In the present survey, the vast majority of organic product consumers (OCOP and RCOP) perceived organic products as being better for health and environment. This is fairly consistent with three previous small-scale surveys [12], [13], [16] and also with a Canadian study indicating that 89% organic food consumers reported nutritional and health motivations [38]. Regarding demographics and socio-economics, we found that a majority of organic product consumers of both genders had a higher education level than the non-consumer clusters, while overall differences in incomes between the clusters of non-consumers and consumers were not striking. However, it is noteworthy that participants in cluster 3, i.e. those who declared that organic food is too expensive, had lower incomes and education levels. In a previous evaluation of organic food consumption patterns in France [13], the authors concluded that the demographic profile of the organic buyer was not related to income, age or family size, but rather to the educational level. In line with our observations, Australian organic food consumers did not show a greater income but had a higher education level [17]. In contrast, in Belgium, organic consumption was positively associated with age and income while a negative association with education was observed [18].

### Food Consumption across Clusters of Organic Product Consumer

We found an overall similarity in daily food consumption in the three clusters of non-consumers**.** In contrast, in both genders, we observed stepwise changes in food group consumption among the clusters of organic product consumers, with marked deviations in the regular consumer cluster (RCOP), and increased consumption of whole grains, vegetables, fruit, soup, dried fruit, legumes, fruit and vegetable juices, sweet products, vegetable oils and nuts. This is in line with a previous observation indicating higher vegetable consumption by organic consumers compared to conventional consumers in Belgium [18]. In addition, lower consumption of meat and processed meat, milk, dairy products, soda, alcoholic beverages, sweets and fat products, added fat and fast foods was observed in organic food consumer clusters. Moreover, the mPNNS-GS, a score reflecting adequacy with dietary guidelines, gradually increases from non-consumer clusters to the OCOP and RCOP clusters. It is noteworthy that consumption of some food groups did not differ between non-consumers and consumers of such organic foods as refined cereals, fish and seafood, cheese and milky desserts, potatoes and tubers and biscuits. The observed plant food-based dietary pattern of organic food consumers, in addition to being closer to the recommended healthy dietary pattern [33], [39], [40], may also better comply with the sustainable diet concept to minimize the environmental impact [1], [41].

### Nutrient Intake across Organic Product Consumer Clusters

Daily intake of energy, total fats, mono-unsaturated fatty acids, phosphorus and calcium did not markedly differ across clusters. In contrast, and consistent with data on food consumption, higher daily intakes by RCOP participants of both genders were found for most minerals and fatty acids, some vitamins and fiber, whereas lower daily intakes of proteins, saturated fatty acids, sodium, vitamin A (retinol), alcohol and cholesterol were found compared to their counterparts. In a study employing simulation analysis for nutrient intake estimation, a higher intake in beta-carotene was found in organic consumers in Belgium [18]. In most cases herein, it was striking to observe that RCOP better fit with French nutritional guidelines [39], [40] than the other groups. That is consistent with our previous finding that the easiest way to attain all nutritional recommendations is to consume more (unrefined) plant foods and less animal, fat-and sugar-rich foods [42].

### Organic Product Consumption and Overweight/Obesity

After accounting for confounding factors, we found that the probability of being overweight or obese was significantly lower in male and female RCOP than in the 3 non-consumer clusters. A significantly reduced probability, but of much less magnitude, was also found in female OCOP. This was probably related to their healthier food pattern, as discussed above. Nevertheless, after further adjustment for the mPNNS-GS score, reflecting the level of adherence to nutritional guidelines, such associations remained. This raises the question of possibly unexplored characteristics also associated with consumption of organic food. Previous research reported markedly lower contamination of organic foods by pesticide residues compared to conventional foods [8]–[11], [43]. Since several studies have reported an association between pesticide exposure or residues in the body and obesity and type 2 diabetes [9], [43]–[46], the possibility of a potential role of organic food in preventing excessive adiposity because of its lower content in pesticide residues should be tested in further studies.

Our study had major strengths, including a web-based platform allowing assessment of accurate dietary data and other types of data [23]–[25], and the large sample size of the Nutrinet-Santé cohort. The use of clustering to separate individuals into mutually exclusive groups can provide a highly accurate description. However, some limitations in the present study should be noted. First, only the frequency, but not the quantity, of actual organic food consumption was available. Secondly, the nutrient intakes were calculated using a single food composition database essentially concerning non-organic products. This likely entailed underestimated nutrient intakes among organic food consumers given the potentially different nutritional composition for some items [9], [11], [43], [47], [48]. Finally, our findings must be interpreted with caution, since most of the NutriNet-Santé participants exhibited a specific socio-economic profile. Indeed, as compared with national estimates [49], our sample included proportionally more women (77.2% versus 52%) and more individuals of high educational level (64.5% versus 24.3% with post-secondary versus primary/secondary education, respectively). This is consistent with existing knowledge regarding the characteristics of participants in volunteer-based studies focusing on nutrition [50].

In conclusion, the present survey of this very large cohort indicated that consumers of organic foods have a higher level of education, a dietary pattern better fitting food-based recommendations and micronutrient/fiber recommended intakes, as well as a sustainable diet concept; moreover, they are less overweight and less obese compared to non-consumers. From a public health standpoint, better knowledge of the characteristics of consumers and non-consumers of organic products is of great importance in promoting behavior aimed at improving the sustainability of the diet. Finally, these findings provide important new insights into organic food consumer profiles, which will be useful for further testing the relationship between organic food intake and health in surveys based on a prospective design such as the Nutrinet-Santé Study.

## Supporting Information

Table S1 **15-point PNNS-GS (Programme National Nutrition Santé-Guidelines score) computation: definition of the 13 components reflecting PNNS recommendations (diet and physical activity), cut-off and scoring.**
(DOCX)

Table S2 **Characteristics of excluded and included participants, NutriNet-Santé (N** = **104, 252).**
(DOCX)

Table S3 **Description of opinions and attitudes (prices, taste, nutritional quality, environment impact, health impact and general opinion) about organic products across the 5 clusters defined according to consumption of organic products, NutriNet-Santé study (N** = **54,311).** Two clusters were composed of consumers of organic products (COP), including regular consumers (cluster 5: RCOP) and occasional consumers (cluster 4: OCOP). Three other clusters grouped individuals who generally did not consume organic products due to the high cost (cluster 3), because they avoided such products (cluster 2) or because they were not interested in organic products.
(DOCX)

## References

[1] (2012) Definition of sustainable diets and Platform for action, final document, In Sustainable diets and Biodiversity, Proceedings of FAO International Scientific Symposium Sustainable diets and Biodiversity united against hunger, Roma, November 3–5, 2010, FAO Edition.

[2] Lairon D (2012) Biodiversity and sustainable nutrition with a food-based approach., In Sustainable diets and Biodiversity, Proceedings of FAO International Scientific Symposium Sustainable diets and Biodiversity united against hunger: Roma, November 3–5, 2010, FAO Edition.

[3] WHO/FAO Expert Consultation (2003) Diet, Nutrition and the Prevention of Chronic Diseases. Geneva: WHO Technical Report.

[4] WHO Europe (2007) The challenge of obesity in the WHO European Region.

[5] Gomiero T, Pimentel D, Paoletti MG (2011) Environmental impact of different agricultural management practices: conventional vs. organic agriculture. Crit Rev Plant Sci 30: 95–124.

[6] Organic World Website. Key Results of “The World of Organic Agriculture 2012”. Internet: www.organic-world.net/yearbook-2011.html. Accessed 2012 September 1.

[7] Organic Trade Organization website. Internet:http://www.ota.com. Accessed 2012 September 1.

[8] Smith-Spangler C, Brandeau ML, Hunter GE, Bavinger JC, Pearson M, et al (2012) Are organic foods safer or healthier than conventional alternatives?: a systematic review. Ann Intern Med 157: 348–366. 22944875 10.7326/0003-4819-157-5-201209040-00007

[9] FSA (Food Standard Agency) (UK) (2009) Comparison of composition (nutrients and other substances) of organically and conventionally produced foodstuffs: a systematic review of available literature.

[10] Benbrook C, McCullum-Gomez C (2009) Organic vs conventional farming. J Am Diet Assoc 109: 809,811. 10.1016/j.jada.2009.03.019 19394464

[11] Lairon D (2010) Nutritional quality and safety of organic food: a review. Agron Sustain Dev 30: 33–41.

[12] van de Vijver LP, van Vliet ME (2012) Health effects of an organic diet-consumer experiences in the Netherlands. J Sci Food Agric 92: 2923–2927. 22331850 10.1002/jsfa.5614

[13] Hassan D, Monier-Dilhan S, Nichele V, Simioni M (2009) Organic Food Consumption Patterns in France. Journal of Agricultural & Food Industrial Organization 7: 1–23.

[14] Hughner RS, McDonagh P, Prothero A, Shultz CJ, Stanton J (2012) Who are organic food consumers? A compilation and review of why people purchase organic food. Journal of Consumer Behaviour 6: 94–110.

[15] Brown E, Dury S, Holdsworth M (2009) Motivations of consumers that use local, organic fruit and vegetable box schemes in Central England and Southern France. Appetite 53: 183–188. 19540288 10.1016/j.appet.2009.06.006

[16] Arvola A, Vassallo M, Dean M, Lampila P, Saba A, et al (2008) Predicting intentions to purchase organic food: the role of affective and moral attitudes in the Theory of Planned Behaviour. Appetite 50: 443–454. 18036702 10.1016/j.appet.2007.09.010

[17] Oates L, Cohen M, Braun L (2012) Characteristics and consumption patterns of Australian organic consumers. J Sci Food Agric 92: 2782–2787. 22473792 10.1002/jsfa.5664

[18] Hoefkens C, Sioen I, Baert K, De MB, De HS, et al (2010) Consuming organic versus conventional vegetables: the effect on nutrient and contaminant intakes. Food Chem Toxicol 48: 3058–3066. 20691244 10.1016/j.fct.2010.07.044

[19] Kahl J, Baars T, Bugel S, Busscher N, Huber M, et al (2012) Organic food quality: a framework for concept, definition and evaluation from the European perspective. J Sci Food Agric 92: 2760–2765. 22407871 10.1002/jsfa.5640

[20] Lairon D, Huber M. (2013) Food quality and possible positive health effects of organic products. Organic farming: prototype for sustainable agriculture? Springer ed. Springer (In press).

[21] Dangour AD, Lock K, Hayter A, Aikenhead A, Allen E, et al (2010) Nutrition-related health effects of organic foods: a systematic review. Am J Clin Nutr 92: 203–210. 20463045 10.3945/ajcn.2010.29269

[22] Hercberg S, Castetbon K, Czernichow S, Malon A, Mejean C, et al (2010) The Nutrinet-Sante Study: a web-based prospective study on the relationship between nutrition and health and determinants of dietary patterns and nutritional status. BMC Public Health 10: 242. 20459807 10.1186/1471-2458-10-242 PMC2881098

[23] Touvier M, Kesse-Guyot E, Mejean C, Pollet C, Malon A, et al (2011) Comparison between an interactive web-based self-administered 24 h dietary record and an interview by a dietitian for large-scale epidemiological studies. Br J Nutr 105: 1055–1064. 21080983 10.1017/S0007114510004617

[24] Vergnaud AC, Touvier M, Mejean C, Kesse-Guyot E, Pollet C, et al (2011) Agreement between web-based and paper versions of a socio-demographic questionnaire in the NutriNet-Sante study. Int J Public Health 56: 407–417. 21538094 10.1007/s00038-011-0257-5

[25] Touvier M, Mejean C, Kesse-Guyot E, Pollet C, Malon A, et al (2010) Comparison between web-based and paper versions of a self-administered anthropometric questionnaire. Eur J Epidemiol 25: 287–296. 20191377 10.1007/s10654-010-9433-9

[26] INSEE (Institut National de la Statistique et des Etudes Economiques) website. Internet: http://www.insee.fr/en/methodes/. Accessed 2012 June 15).

[27] Craig CL, Marshall AL, Sjostrom M, Bauman AE, Booth ML, et al (2003) International physical activity questionnaire: 12-country reliability and validity. Med Sci Sports Exerc 35: 1381–1395. 12900694 10.1249/01.MSS.0000078924.61453.FB

[28] Hallal PC, Victora CG (2004) Reliability and validity of the International Physical Activity Questionnaire (IPAQ). Med Sci Sports Exerc 36: 556. 15076800 10.1249/01.mss.0000117161.66394.07

[29] Hagstromer M, Oja P, Sjostrom M (2006) The International Physical Activity Questionnaire (IPAQ): a study of concurrent and construct validity. Public Health Nutr 9: 755–762. 16925881 10.1079/phn2005898

[30] Le Moullec N, Deheeger M, Preziosi P, Montero P, Valeix P, et al (1996) Validation du manuel photos utilisé pour l’enquête alimentaire de l’étude SU.VI.MAX. Cahier de Nutrition et de Diététique 31: 158–164.

[31] Black AE (2000) Critical evaluation of energy intake using the Goldberg cut-off for energy intake:basal metabolic rate. A practical guide to its calculation, use and limitations. Int J Obes Relat Metab Disord 24: 1119–1130. 11033980 10.1038/sj.ijo.0801376

[32] Schofield WN (1985) Predicting basal metabolic rate, new standards and review of previous work. Hum Nutr Clin Nutr 39 Suppl 15–41. 4044297

[33] Chauliac M, Razanamahefa L, Choma C, Boudot J, Houssin D (2009) National health and nutrition program: challenges of a global action plan. Rev Prat 59: 10–12. 19253871

[34] Estaquio C, Kesse-Guyot E, Deschamps V, Bertrais S, Dauchet L, et al (2009) Adherence to the French Programme National Nutrition Sante Guideline Score is associated with better nutrient intake and nutritional status. J Am Diet Assoc 109: 1031–1041. 19465185 10.1016/j.jada.2009.03.012

[35] Khattree R, Naik DN (2000) Multivariate Data Reduction and Discrimination With Sas Software.

[36] Der G, Everitt B (2002) A Handbook of Statistical Analyses using SAS. London: Chapman & Hall/CRC.

[37] Hosmer DW, Lesmeshow S (2000) Applied Logistic Regression. New York: Wiley Series in Probability and Mathematical Statistics.

[38] Agriculture and rural development ministry website. Organic Consumer Profile. Internet: http://www.agric.gov.ab.ca/food/organic/organic_profile.html. Accessed 2012 September 1.

[39] CNERNA CNRS (2000) Apports nutritionnels conseillés pour la population française. Paris: Tec & Doc Lavoisier.

[40] Legrand P, Morise A, Kalonji E (2011) Update of French nutritional recommendations for fatty acids. World Rev Nutr Diet 102: 137–143. 21865827 10.1159/000327800

[41] Macdiarmid JI, Kyle J, Horgan GW, Loe J, Fyfe C, et al (2012) Sustainable diets for the future: can we contribute to reducing greenhouse gas emissions by eating a healthy diet? Am J Clin Nutr 96: 632–639. 22854399 10.3945/ajcn.112.038729

[42] Maillot M, Issa C, Vieux F, Lairon D, Darmon N (2011) The shortest way to reach nutritional goals is to adopt Mediterranean food choices: evidence from computer-generated personalized diets. Am J Clin Nutr 94: 1127–1137. 21900460 10.3945/ajcn.111.016501

[43] AFSSA (French food safety agency) (2003) Report on evaluation of the nutritional and sanitary quality of organic foods (Evaluation nutritionnelle et sanitaire des aliments issus de l’agriculture biologique, in French).

[44] Dirinck E, Jorens PG, Covaci A, Geens T, Roosens L, et al (2011) Obesity and persistent organic pollutants: possible obesogenic effect of organochlorine pesticides and polychlorinated biphenyls. Obesity (Silver Spring) 19: 709–714. 20559302 10.1038/oby.2010.133

[45] Thayer KA, Heindel JJ, Bucher JR, Gallo MA (2012) Role of environmental chemicals in diabetes and obesity: a National Toxicology Program workshop review. Environ Health Perspect 120: 779–789. 22296744 10.1289/ehp.1104597 PMC3385443

[46] Lee DH, Steffes MW, Sjodin A, Jones RS, Needham LL, et al (2011) Low dose organochlorine pesticides and polychlorinated biphenyls predict obesity, dyslipidemia, and insulin resistance among people free of diabetes. PLoS One 6: e15977. 21298090 10.1371/journal.pone.0015977 PMC3027626

[47] Brandt K, Leifert C, Sabderson R, Seal CJ (2011) Agroecosystem management and nutritional quality of plant foods: the case of organic fruits and vegetables. Crit Rev Plant Sci 30: 197.

[48] Rembialkowska E (2007) Quality of plant products from organic agriculture. Journal of the Science of Food and Agriculture 87: 2757–2762.

[49] INSEE (Institut National de la Statistique et des Etudes Economiques) website. French national census data. Internet: http://www.insee.fr/fr/bases-de-donnees/default.asp?page=recensements.htm. Accessed 2012 June 15.

[50] Rothman KJ, Greenland S, Lash TL (2008) Modern epidemiology. 2nd edition ed. Philadelphia: Lippincott Williams and Wilkins.

