# Supplementary material for: Profiles of Organic Food Consumers in a Large Sample of French Adults: Results from the Nutrinet-Santé Cohort Study
Source: PLoS One. 2013 Oct 18;8(10):e76998. doi: 10.1371/journal.pone.0076998 (PMC3800052; doi:10.1371/journal.pone.0076998)
Supplement: Table S1 — 15-point PNNS-GS (Programme National Nutrition Santé-Guidelines score) computation: definition of the 13 components reflecting PNNS recommendations (diet and physical activity), cut-off and scoring. (DOCX) [file pone.0076998.s001.docx]

Supplementary Table 1. PNNS-GS: components and scores according to PNNS recommendations

|  | Recommendation | Scoring criteria^1^ | Score |
| --- | --- | --- | --- |
| **Fruit and vegetables** | At least 5/d | [0-3.5[ | 0 |
|  |  | [3.5-5[ | 0.5 |
|  |  | [5-7.5[ | 1 |
|  |  | ≥7.5 | 2 |
| **Bread, cereals, potatoes and legumes** | At each meal according to appetite | [0-1[ | 0 |
|  |  | [1-3[ | 0.5 |
|  |  | [3-6[ | 1 |
|  |  | ≥6 | 0.5 |
| **Whole grain food** | Choose whole grains and whole-grain breads more often | [0-1/3[ | 0 |
|  |  | [1/3-2/3[ | 0.5 |
|  |  | ≥2/3 | 1 |
| **Milk and dairy products** | 3/d (≥55-years-old: 3 to 4/d) | [0-1[ | 0 |
|  |  | [1-2.5[ | 0.5 |
|  |  | [2.5-3.5] (55-years-old: [2.5-4.5]) | 1 |
|  |  | >3.5 (55-years-old : >4.5) | 0 |
| **Meat, poultry seafood and eggs** | 1 to 2/d | 0 | 0 |
|  |  | ]0-1[ | 0.5 |
|  |  | [1-2] | 1 |
|  |  | >2 | 0.5 |
| **Seafood** | At least 2/week | < 2/week | 0 |
|  |  | ≥ 2/week | 1 |
| **Added fat** | Limit consumption | Lipids from added fat >16% EI^3^/d | 0 |
|  |  | Lipids from added fat ≤16% EI/d | 1 |
| **Vegetable added fat** | Favor fat of vegetable origin | No use of vegetable oil or ratio vegetable oil/total added fats≤0.5 | 0 |
|  |  | No use of added fats or ratio of vegetable oil/total added fats >0.5 | 1 |
| **Sweetened foods** | Limit consumption | Added sugar from sweetened foods ≥17.5% EI/d | -0.5 |
|  |  | Added sugar from sweetened foods 17.5-12.5% EI/d | 0 |
|  |  | Added sugar from sweetend foods <12.5% EI/d | 1 |
| **Beverages** |  |  |  |
| **Non-alcoholic beverages** | Drink water as desired  Limit sweetened beverages: no more than 1 glass/d | <1l water and >250 ml soda/d | 0 |
|  |  | ≥1l water and >250 ml soda/d | 0.5 |
|  |  | <1 l water and ≤250 ml soda/d | 0.75 |
|  |  | ≥1 l water and ≤250 ml soda/d | 1 |
| **Alcohol** | Women advised to drink ≤2 glasses of wine/d and men ≤3 glasses/d | Ethanol >20 g/d for women and >30 g/d for men | 0 |
|  |  | Ethanol ≤20 g/d for women and ≤30 g/d for men | 0.8 |
|  |  | Abstainers and irregular consumers (<once a week) | 1 |
| **Salt^2^** | Limit consumption | >12g/d | -0.5 |
|  |  | ]10-12] g/d | 0 |
|  |  | ]8-10] g/d | 0.5 |
|  |  | ]6-8] g/d | 1 |
|  |  | ≤6 g/d | 1.5 |
| **Physical activity** | At least the equivalent of 30 min/d of brisk walking | [0-30(min/d | 0 |
|  |  | [30-60(min/d | 1 |
|  |  | ≥60 min/d | 1.5 |

^1^Servings per day unless otherwise indicated

^2^ Established according to French recommended dietary allowances

^3^ EI: energy intake without alcohol

Supplementary Table 2. Characteristics of excluded and included participants, NutriNet-Santé (N=104, 252)^1^

|  | Included | Excluded | P |
| --- | --- | --- | --- |
| N | 54,311 | 49,941 |  |
| **Age (y)** | 43.7 (14.4) | 42.1 (14.4) | <0.0001 |
| **BMI (kg/m²)** | 23.8 (4.5) | 24.3 (4.9) | <0.0001 |
| **Education (%)** |  |  | <0.0001 |
| ≤ High school diploma | 18.7 | 21.8 |  |
| High school | 16.8 | 18.6 |  |
| Post-secondary graduate | 64.5 | 59.6 |  |
| **Physical activity (%)** |  |  | <0.0001 |
| Low | 23.3 | 25.0 |  |
| Medium | 42.5 | 41.2 |  |
| High | 34.1 | 33.8 |  |
| **Tobacco use** **(%)** |  |  | <0.0001 |
| Never-smokers | 49.8 | 47.8 |  |
| Former smokers | 34.0 | 32.0 |  |
| Current smokers | 16.2 | 20.2 |  |

^1^P values referred to chi-square test or t-test

Supplementary Table 3. Opinions and attitudes about organic products across clusters compared to non-organic products

| **%** | Cluster 1 | Cluster 2 | Cluster 3 | Cluster 4 | Cluster 5 | Total |
| --- | --- | --- | --- | --- | --- | --- |
|  | Not interested | Avoidance | Too expensive | OCOP | RCOP |  |
| ***What is your opinion about?*** |  |  |  |  |  |  |
| **Prices of organic products** |  |  |  |  |  |  |
| Expensive -will not buy them | 61.7 | 67.1 | 98.6 | 49.8 | 2.3 | 51.0 |
| Expensive –but will buy them | 10.3 | 7.0 | 0.6 | 36.3 | 73.2 | 31.1 |
| Price is similar | 5.8 | 4.1 | 0.2 | 8.8 | 22.2 | 9.0 |
| Less expensive | 0.1 | 0.2 | 0.1 | 0.0 | 0.2 | 0.1 |
| No opinion | 22.1 | 21.6 | 0.5 | 5.0 | 2.2 | 8.8 |
| **Nutritional quality** |  |  |  |  |  |  |
| Less healthy | 0.2 | 1.6 | 0.4 | 0.4 | 1.3 | 0.6 |
| Sometimes less healthy | 2.7 | 3.8 | 2.1 | 2.6 | 1.7 | 2.6 |
| Equivalent | 47.3 | 37.4 | 34.3 | 34.6 | 19.5 | 34.9 |
| Sometimes more healthy | 15.8 | 12.2 | 16.2 | 22.6 | 18.8 | 19.3 |
| More healthy | 10.4 | 11.9 | 25.5 | 31.2 | 56.2 | 28.8 |
| No opinion | 23.5 | 33.1 | 21.6 | 8.5 | 2.6 | 13.8 |
| **Taste** |  |  |  |  |  |  |
| Less tasty | 0.8 | 2.7 | 0.8 | 0.6 | 0.7 | 0.9 |
| Sometimes less tasty | 7.5 | 6.9 | 5.6 | 7.9 | 4.1 | 7.0 |
| Equivalent | 35.9 | 28.0 | 28.0 | 27.9 | 13.2 | 27.2 |
| Sometimes better | 17.4 | 11.2 | 16.3 | 27.0 | 25.1 | 22.6 |
| Better | 10.2 | 10.5 | 19.1 | 29.3 | 56.0 | 27.1 |
| No opinion | 28.3 | 40.6 | 30.2 | 7.3 | 1.0 | 15.3 |
| **Health impact** |  |  |  |  |  |  |
| Worse | 0.8 | 1.4 | 1.0 | 1.1 | 1.1 | 1.1 |
| No influence | 27.3 | 28.8 | 15.4 | 8.6 | 1.5 | 13.4 |
| Better | 43.3 | 36.2 | 62.5 | 79.8 | 95.6 | 69.9 |
| I don't know | 28.6 | 33.7 | 21.2 | 10.6 | 1.8 | 15.6 |
| **Environmental impact** |  |  |  |  |  |  |
| Worse | 2.3 | 2.6 | 2.3 | 2.0 | 1.2 | 2.0 |
| No influence | 8.4 | 12.8 | 5.7 | 3.1 | 0.8 | 4.9 |
| Better | 72.7 | 60.1 | 78.3 | 89.4 | 97.1 | 83.7 |
| I don't know | 16.6 | 24.6 | 13.7 | 5.4 | 1.0 | 9.4 |
| **General opinion about organic products** |  |  |  |  |  |  |
| Marketing techniques overrate them | 33.3 | 33.8 | 29.2 | 19.5 | 7.1 | 23.1 |
| No opinion | 7.1 | 6.9 | 2.8 | 1.4 | 0.1 | 3.0 |
| Wary of them | 9.6 | 14.7 | 10.6 | 5.8 | 1.5 | 7.6 |
| Valuable product in the future | 18.2 | 15.1 | 26.7 | 40.4 | 66.6 | 34.9 |
| Not enough information | 27.8 | 26.1 | 27.3 | 29.2 | 20.4 | 27.7 |
| No response | 4.0 | 3.4 | 3.5 | 3.7 | 4.5 | 3.8 |
