# Supplementary material for: Profiles of Organic Food Consumers in a Large Sample of French Adults: Results from the Nutrinet-Santé Cohort Study
Source: PLoS One. 2013 Oct 18;8(10):e76998. doi: 10.1371/journal.pone.0076998 (PMC3800052; doi:10.1371/journal.pone.0076998)
Supplement: Table S2 — Characteristics of excluded and included participants, NutriNet-Santé (N = 104, 252). (DOCX) [file pone.0076998.s002.docx]

Supplementary Table 2. Characteristics of excluded and included participants, NutriNet-Santé (N=104, 252)^1^

|  | Included | Excluded | P |
| --- | --- | --- | --- |
| N | 54,311 | 49,941 |  |
| **Age (y)** | 43.7 (14.4) | 42.1 (14.4) | <0.0001 |
| **BMI (kg/m²)** | 23.8 (4.5) | 24.3 (4.9) | <0.0001 |
| **Education (%)** |  |  | <0.0001 |
| ≤ High school diploma | 18.7 | 21.8 |  |
| High school | 16.8 | 18.6 |  |
| Post-secondary graduate | 64.5 | 59.6 |  |
| **Physical activity (%)** |  |  | <0.0001 |
| Low | 23.3 | 25.0 |  |
| Medium | 42.5 | 41.2 |  |
| High | 34.1 | 33.8 |  |
| **Tobacco use** **(%)** |  |  | <0.0001 |
| Never-smokers | 49.8 | 47.8 |  |
| Former smokers | 34.0 | 32.0 |  |
| Current smokers | 16.2 | 20.2 |  |

^1^P values referred to chi-square test or t-test
