# Supplementary material for: Profiles of Organic Food Consumers in a Large Sample of French Adults: Results from the Nutrinet-Santé Cohort Study
Source: PLoS One. 2013 Oct 18;8(10):e76998. doi: 10.1371/journal.pone.0076998 (PMC3800052; doi:10.1371/journal.pone.0076998)
Supplement: Table S3 — Description of opinions and attitudes (prices, taste, nutritional quality, environment impact, health impact and general opinion) about organic products across the 5 clusters defined according to consumption of organic products, NutriNet-Santé study (N = 54,311). Two clusters were composed of consumers of organic products (COP), including regular consumers (cluster 5: RCOP) and occasional consumers (cluster 4: OCOP). Three other clusters grouped individuals who generally did not consume organic products due to the high cost (cluster 3), because they avoided such products (cluster 2) or because they were not interested in organic products. (DOCX) [file pone.0076998.s003.docx]

Supplementary Table 3. Opinions and attitudes about organic products across clusters

| **%** | Cluster 1 | Cluster 2 | Cluster 3 | Cluster 4 | Cluster 5 | Total |
| --- | --- | --- | --- | --- | --- | --- |
|  | Not interested | Avoidance | Too expensive | OCOP | RCOP |  |
| ***What is your opinion about?*** |  |  |  |  |  |  |
| **Prices of organic products** |  |  |  |  |  |  |
| Expensive -will not buy them | 61.7 | 67.1 | 98.6 | 49.8 | 2.3 | 51.0 |
| Expensive –but will buy them | 10.3 | 7.0 | 0.6 | 36.3 | 73.2 | 31.1 |
| Price is similar | 5.8 | 4.1 | 0.2 | 8.8 | 22.2 | 9.0 |
| Less expensive | 0.1 | 0.2 | 0.1 | 0.0 | 0.2 | 0.1 |
| No opinion | 22.1 | 21.6 | 0.5 | 5.0 | 2.2 | 8.8 |
| **Nutritional quality** |  |  |  |  |  |  |
| Less healthy | 0.2 | 1.6 | 0.4 | 0.4 | 1.3 | 0.6 |
| Sometimes less healthy | 2.7 | 3.8 | 2.1 | 2.6 | 1.7 | 2.6 |
| Equivalent | 47.3 | 37.4 | 34.3 | 34.6 | 19.5 | 34.9 |
| Sometimes more healthy | 15.8 | 12.2 | 16.2 | 22.6 | 18.8 | 19.3 |
| More healthy | 10.4 | 11.9 | 25.5 | 31.2 | 56.2 | 28.8 |
| No opinion | 23.5 | 33.1 | 21.6 | 8.5 | 2.6 | 13.8 |
| **Taste** |  |  |  |  |  |  |
| Less tasty | 0.8 | 2.7 | 0.8 | 0.6 | 0.7 | 0.9 |
| Sometimes less tasty | 7.5 | 6.9 | 5.6 | 7.9 | 4.1 | 7.0 |
| Equivalent | 35.9 | 28.0 | 28.0 | 27.9 | 13.2 | 27.2 |
| Sometimes better | 17.4 | 11.2 | 16.3 | 27.0 | 25.1 | 22.6 |
| Better | 10.2 | 10.5 | 19.1 | 29.3 | 56.0 | 27.1 |
| No opinion | 28.3 | 40.6 | 30.2 | 7.3 | 1.0 | 15.3 |
| **Health impact** |  |  |  |  |  |  |
| Worse | 0.8 | 1.4 | 1.0 | 1.1 | 1.1 | 1.1 |
| No influence | 27.3 | 28.8 | 15.4 | 8.6 | 1.5 | 13.4 |
| Better | 43.3 | 36.2 | 62.5 | 79.8 | 95.6 | 69.9 |
| I don't know | 28.6 | 33.7 | 21.2 | 10.6 | 1.8 | 15.6 |
| **Environmental impact** |  |  |  |  |  |  |
| Worse | 2.3 | 2.6 | 2.3 | 2.0 | 1.2 | 2.0 |
| No influence | 8.4 | 12.8 | 5.7 | 3.1 | 0.8 | 4.9 |
| Better | 72.7 | 60.1 | 78.3 | 89.4 | 97.1 | 83.7 |
| I don't know | 16.6 | 24.6 | 13.7 | 5.4 | 1.0 | 9.4 |
| **General opinion about organic products** |  |  |  |  |  |  |
| Marketing techniques overrate them | 33.3 | 33.8 | 29.2 | 19.5 | 7.1 | 23.1 |
| No opinion | 7.1 | 6.9 | 2.8 | 1.4 | 0.1 | 3.0 |
| Wary of them | 9.6 | 14.7 | 10.6 | 5.8 | 1.5 | 7.6 |
| Valuable product in the future | 18.2 | 15.1 | 26.7 | 40.4 | 66.6 | 34.9 |
| Not enough information | 27.8 | 26.1 | 27.3 | 29.2 | 20.4 | 27.7 |
| No response | 4.0 | 3.4 | 3.5 | 3.7 | 4.5 | 3.8 |
